# Malignant perivascular epithelioid cell tumor (PEComa) of the femur: a case report and literature review

**DOI:** 10.1186/s13000-015-0292-2

**Published:** 2015-05-29

**Authors:** I Weng Lao, Lin Yu, Jian Wang

**Affiliations:** Department of Pathology, Fudan University Shanghai Cancer Center, 270 Dong An Street, Shanghai, 200032 China; Department of Oncology, Shanghai Medical College, Fudan University, 270 Dong An Street, Shanghai, 200032 China

**Keywords:** Perivascular epithelioid cell tumor (PEComa), Bone neoplasm, Immunohistochemistry

## Abstract

**Background:**

We describe a case of malignant perivascular epithelial cell tumor (PEComa) arising primarily in the distal left femur of a 47-year-old male.

**Case presentation:**

The patient presented with pain accompanied by progressive swelling of his left thigh. Computed tomography (CT) scan and magnetic resonance imaging (MRI) revealed an osteolytic lesion. Curettage of the lesion was reported as a clear cell tumor with recommendation for exclusion of a metastatic clear cell carcinoma. However, thorough examinations did not find any primary site elsewhere, apart from the presence of bilateral pulmonary metastases. Evaluation of the submitted H & E slides identified a malignant PEComa which was further confirmed by subsequent immunohistochemical study.

**Conclusions:**

The occurrence of PEComa as a primary bone lesion is extremely rare. We present here a malignant PEComa of the distal left femur, and summarize the clinicopathological characteristics of this rare entity with literature review.

**Virtual slides:**

The virtual slide (s) for this article can be found here: http://www.diagnosticpathology.diagnomx.eu/vs/5729035221600545.

**Electronic supplementary material:**

The online version of this article (doi:10.1186/s13000-015-0292-2) contains supplementary material, which is available to authorized users.

## Background

Perivascular epithelioid cell tumor (PEComa) represents a family of closely related entities showing both melanocytic and myoid differentiation, including angiomyolipoma, lymphangioleiomyomatosis, clear-cell ‘sugar’ tumor of the lung, and neoplasms arising in a wide variety of locations including skin, soft tissue and visceral organs called PEComa not otherwise specified (PEComa-NOS) [1]. PEComa manifesting as a primary bone lesion is extremely rare. To date, only 10 convincing cases of primary bone origin have been reported in the English literature [2–8]. Although the majority of PEComas behave in a benign or indolent fashion, a minority of tumors exhibit aggressive behavior. The malignant variant may cause diagnostic pitfalls, particularly in interpretation of biopsy specimens. In this report, we describe a case of malignant PEComa arising primarily in the femur to broaden the anatomic spectrum of primary bone PEComas.

## Case presentation

### Clinical presentation

**Fig. 1:**
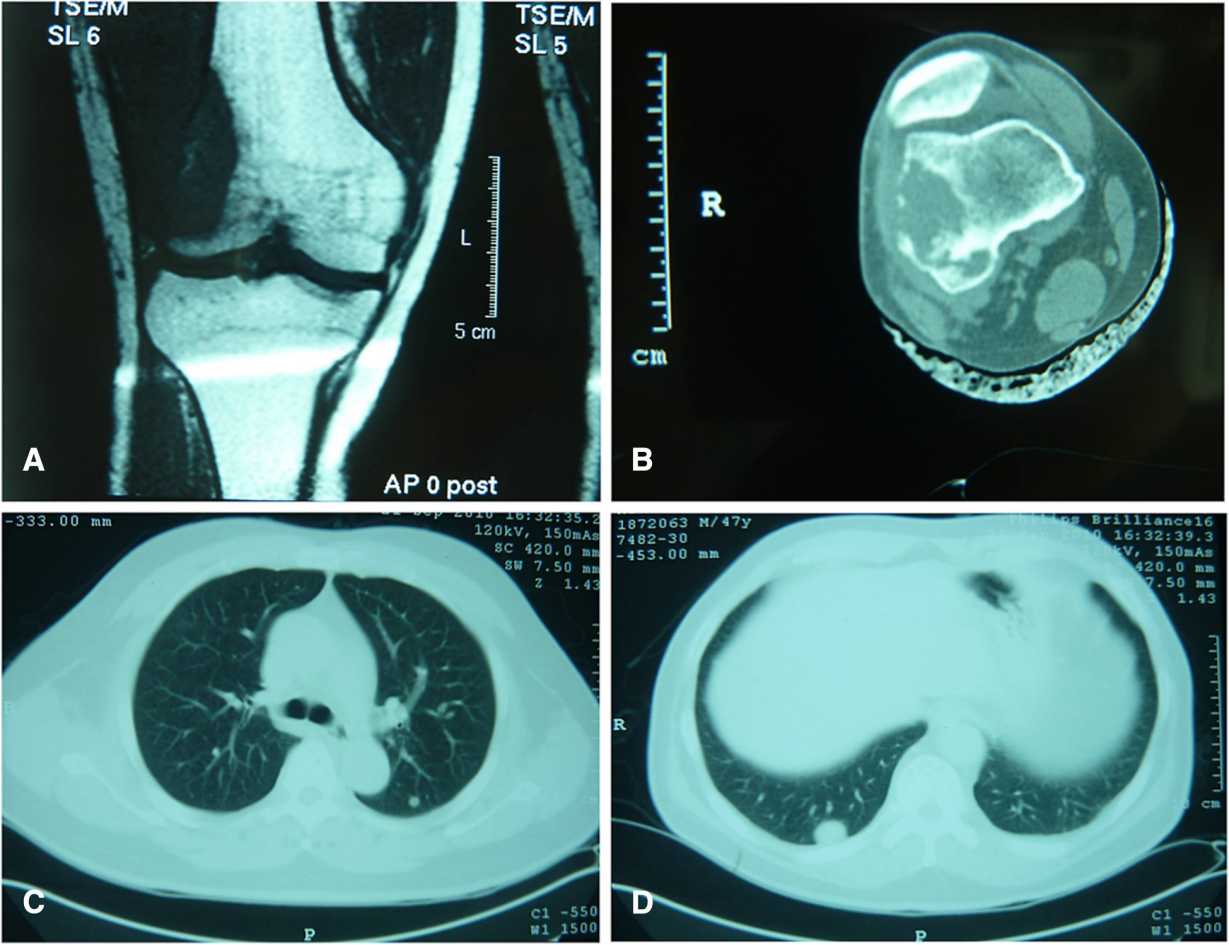
Radiology. Magnetic resonance imaging scan shows an osteolytic lesion in the distal femur (**a**). CT scan demonstrates cortical destruction and extension into adjacent soft tissue (**b**). Chest CT shows metastatic disease in bilateral lungs (**c,d**)

A 47-year-old male presented with a 2-year history of pain and progressive swelling of his left thigh. Computed tomography (CT) scan and magnetic resonance imaging (MRI) of the left lower limb revealed an osteolytic lesion in the distal femur (Figs. 1a and b), measuring 5.2 × 3.2 × 2.6 cm in size. The lesion showed destruction of the cortex with extension into the surrounding skeletal muscle. Curettage of the lesion was performed which was initially reported as a clear cell tumor with recommendation for the exclusion of a metastatic clear cell carcinoma. No evidence of a primary tumor in any site elsewhere was identified by subsequent systematic examinations. However, CT scan of the chest revealed multiple nodules in bilateral lungs which were confirmed as metastatic by CT-guided core needle biopsy (Figs. 1c and d). The patient was treated with conservative palliative radiotherapy and systemic chemotherapy. He was alive with the disease at 3.5 year follow up.

### Pathological findings

**Fig. 2:**
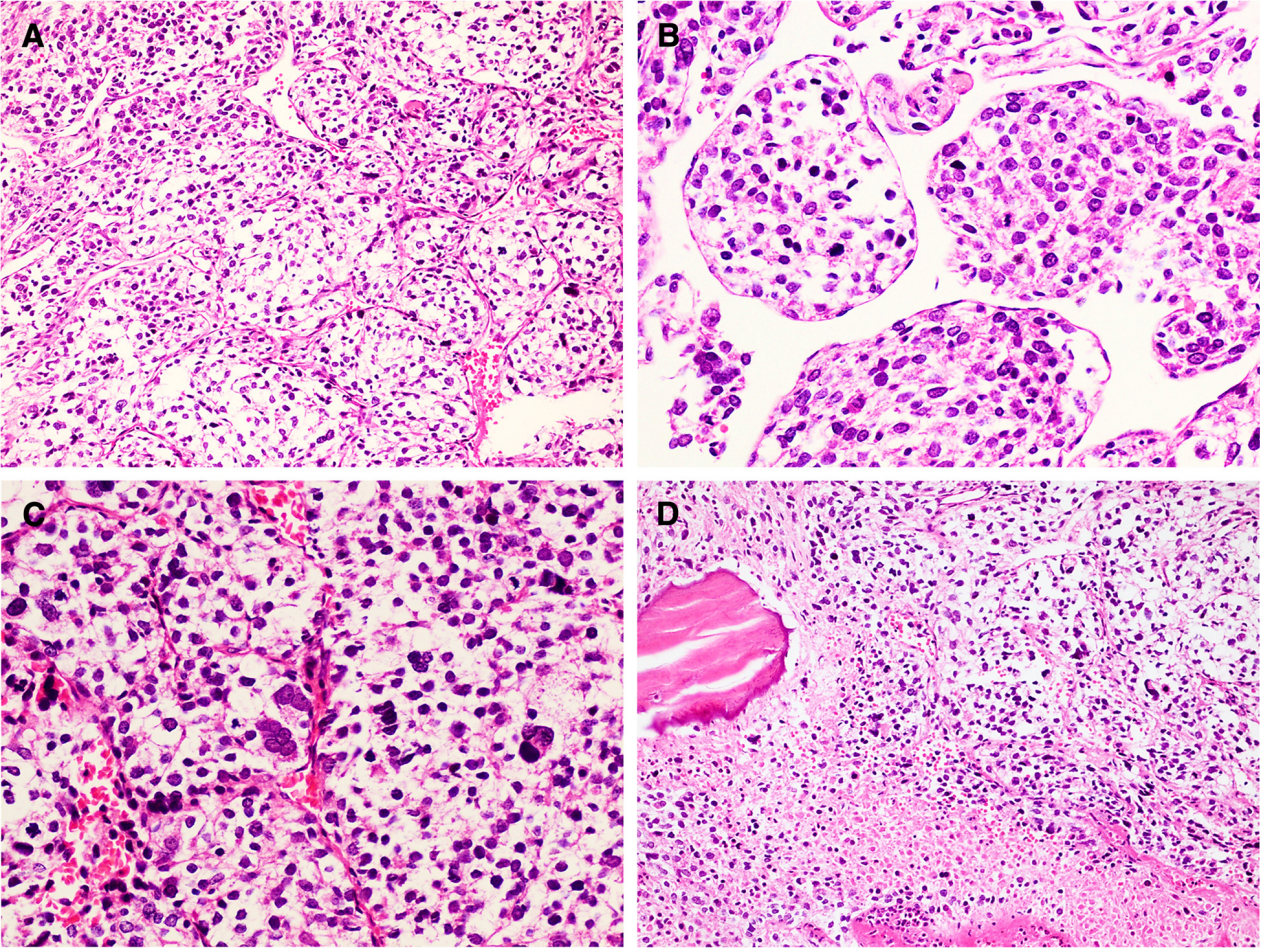
Histology. Epithelioid cells arranged in nested pattern with delicate arborizing vasculature (×100, **a**). Sinusoidal-type vasculature in PEComa (×200, **b**). Nuclear pleomorphism with multinucleate tumor cells and mitotic activity (×200, **c**). Area of necrosis present within the tumor (×100, **d**)

**Fig. 3:**
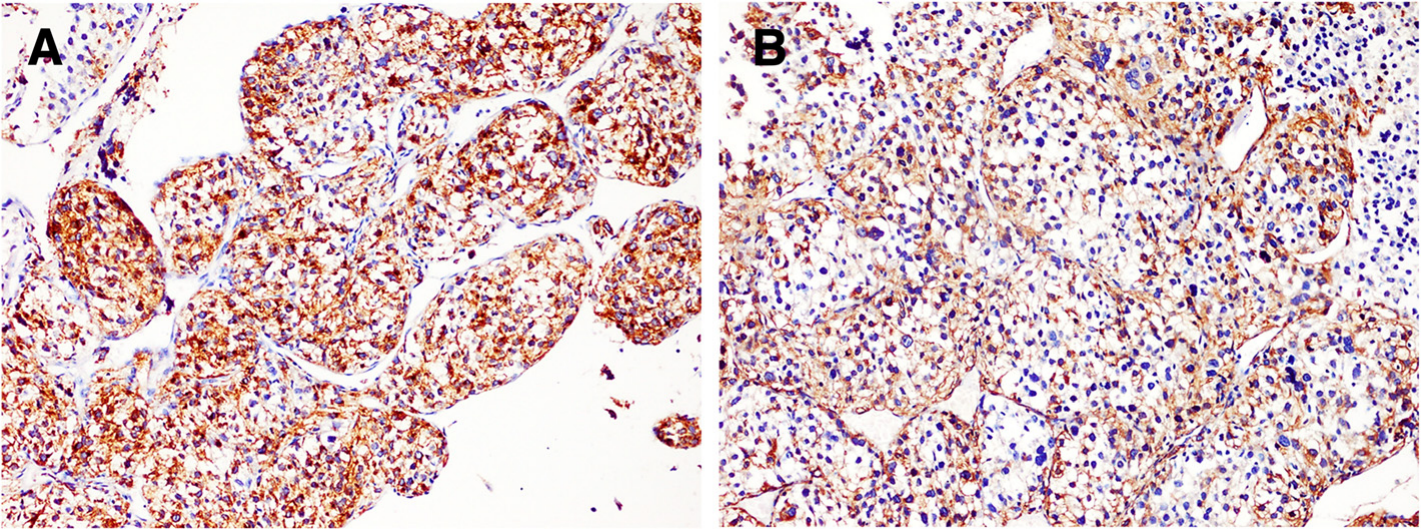
Immunohistochemistry. HMB45 (EnVision × 100, **a**). Alpha smooth muscle actin (EnVision × 100, **b**)

Histologically, the tumor was composed of plump polygonal cells with abundant clear to palely eosinophilic or granular cytoplasm arranged in nests that were separated by a delicate aborizing or sinusoidal-type vasculature (Figs. 2a and b). The tumor cells showed a moderate to high degree of hypercellularity and nuclear atypia (Fig. 2c). A few multinucleated giant cells were also observed. Mitotic figures were readily encountered (5/50HPF). Occasional atypical mitotic figures were present. Areas of coagulative tumor necrosis were obvious within the tumor (Fig. 2d). Immunohistochemically, the tumor cells were strongly positive for HMB45 (Fig. 3a), PNL2, TFE3 and vimentin. Most tumor cells were also positive for alpha smooth muscle actin (Fig. 3b). There was weak staining of CD10 and CD117. CD34 staining clearly delineated the rich vascular network, highlighting the organoid or sinusoidal architecture. The tumor cells were negative for Melan-A, AE1/AE3, EMA, desmin and S100 protein.

## Discussions

The first case of primary bone PEComa was described by Insabato et al. in 2002 which occurred in the tibia of a 30-year-old male [2]. The presentation of PEComa as a primary bone lesion is extremely rare. Taking the current case into account, there are only 11 cases of primary bone origin. The clinical features of these 11 primary bone PEComas are summarized in Table 1. The site of involvement includes right tibia in 3 cases [2, 6], right fibula in 3 cases [3, 4, 8], vertebral column in 2 cases (7th thoracic and 5th lumbar respectively) [6, 7], the sixth right rib [5], left acetabulum [8], and left femur (current case) in 1 case each. Like PEComas arising in other sites, primary bone PEComas also occurred in middle-aged adults with a mean age of 47 years and median age of 39 years (range, 26 to 93 years) respectively. Unlike PEComas of other sites which tended to have a female predilection, primary bone PEComas were distributed approximately equally in both sexes. This disparity is possibly due to the limited number of bone PEComas being reported thus far. In accordance with the very low frequency of TSC-associated PEComas, none of the patients with bone PEComas had association with TSC.

**Table 1:** Clinical features of 11 cases of primary bone PEComa

Author/Reference	Age (y)/Sex	Site	Size (cm)	Presentation	Radiologic features	Treatment	Follow-up
Insabato et al. [2]	30/M	Right proximal tibia	2	Pain	Osteolytic with cortical destruction	Local resection	ANED, 12mo
Torri et al. [5]	28/M	Right 6th rib	2	Pain	Osteolytic	Complete resection	ANED, not reported
Righi et al. [4]	92/F	Right fibula	NA	NA	NA	Local resection	NA
Lian et al. [3]	52/F	Right mid-shaft fibula	6.3	Progressive swelling	Extension through the cortex forming a soft-tissue mass	Wide excision	ANED, 3mo
Yamashita et al. [6], case 1	35/M	7th thoracic vertebra	1.8	Bilateral leg weakness, back pain	Osteolytic, destructive enhancing lesion (MRI)	CRT	Pelvic bone metastases, AWD,12mo
Yamashita et al. [6], case 2	39/F	Right tibia	6.5	Pain	Enhancing mass with areas of breakthrough of the cortex forming a soft-tissue mass	RT + Excision	ANED,34mo
Yamashita et al. [6], case 3	48/F	Right distal tibia	Very small	Pain	Permeable destructive lesion with soft tissue extension (recurrent lesion)	Excisional biopsy, amputation	Recurred 3 times in 3 y, ANED, 3y
Kazzaz et al. [7]	26/M	5th lumbar vertebra	Large	Lower back pain, left leg weakness	Destructive lesion with extra-osseous mass	Conservative	Lung metastases, ANED, not reported
Desy et al. [8], case 1	93/F	Right distal fibula	NA	Progressive pain, swelling	Expansile lytic lesion	Local resection	ANED,2y
Desy et al. [8], case 2	29/M	Left acetabulum	5	Progressive hip pain	Extensive lytic with soft tissue expansion	Left hemipelvectomy + temsirolimus	Lung metastases, DOD, 8mo
Lao et al. (our case)	47/M	Left femur	5.2	Pain, progressive swelling	Osteolytic mass, destruction of cortex forming a soft-tissue mass	Curettage + CRT	Lung metastases, AWD, 3.5y

*ANED* indicates alive with no evidence of disease, *AWD* alive with disease, *CRT* chemo-radiotherapy, *RT, radiotherapy*, *DOD* died of disease, *mo* month, NA, not available *y* year

Clinically, patients with bone PEComas typically presented with pain. Three patients were accompanied with swelling [3, 8], one of whom had a pathologic fracture of the distal fibula [8]. One patient was accompanied with bilateral leg weakness because of cord compression [6]. Radiologically, primary PEComas of bone frequently appeared as osteolytic lesions. In more aggressive cases, destruction of cortex with forming of soft tissue mass could be noted [2, 6–8]. On MR imaging, the tumor was usually hypointense on T1-weighted imaging and hyperintense on T2-weighted images [6, 8]. In a biopsy-proven malignant PEComa, F-18 FDG PET demonstrated intense hypermetabolism [9]. Of note, FDG PET scans were often negative in patients with benign PEComas and positive in malgiant PEComas [10]. However, a benign PEComa of the lungs with extensive FDG uptake has also been described [11].

Histologically, most bone PEComas were composed of epithelioid perivascular cells that exhibited characteristic nesting or organoid arrangement. Two case were composed of both epithelioid and spindle cells [6]. Of 11 cases, 3 were considered to be benign which were also supported by the clinical outcome [2, 4, 6, 8]. One case was supposed to have malignant potential based on the invasion into the surrounding connective tissues and overexpression of cyclinD1 [5]. The remaining 7 cases were recognized histologically as fully malignant. Besides permeative growth pattern, marked nuclear atypia was present in all 7 cases, necrosis was seen in 2 cases (including the current case) [3], and mitotic activity was noted in 6 cases, ranging from 5/50HPF to 36/50HPF [6]. Vascular invasion was identified in 2 cases [3, 6]. It is worthy to note that although most cases of malignant PEComa fulfilled the morphological criteria for malignancy proposed by Folpe et al. [1], rare example existed which was not clearly malignant on histological ground, especially on biopsy specimens [7]. In such instance, the malignancy was usually betrayed by its aggressive clinical behaviour.

The major differential diagnosis in the current case includes metastatic clear cell carcinoma, especially of renal origin. However, immunohistochemical study ruled out this possibility as the tumor cells failed to express epithelial markers. Alveolar soft part sarcoma (ASPS), a sarcoma characterized by organoid pattern and sinusoidal-type vasculature, may cause confusion with PEComa. In particular, both tumors can express TFE3 [4, 12]. The absence of melanocytic differentiation in ASPS is helpful in the separation of these two entities. The other lesions that may enter the differential diagnoses are metastatic malignant melanoma and clear cell sarcoma. In addition to morphological differences, strong S100 protein and negative staining of myogenic markers in most melanomas and clear cell sarcomas (CCS), the presence of specific EWSR1 ATF1 fusion transcripts resulting from t (12:22) (q13; q12) in CCS are helpful features to distinguish the lesions from PEComa.

It seems that malignant PEComa of bone has a tendency to develop metastatic disease. Of 7 malignant bone PEComas, four developed metastatic disease, including pelvic bone metastases in one case [6], and lung metastases in other 3 cases (including the current case) [7, 8]. At present, the optimal therapy for malignant PEComa remains a challenging practice. The activation of the mTOR signaling pathway was thought mechanistically to be linked to the development of both TSC-associated and non-TSC-associated PEComa [13]. Based on this pathologic mechanism, mTOR inhibitors (sirolimus/rapamycin) was applied in small number of cases and considered to be a rational molecular target for therapy in malignant PEComa [14]. One patient with a malignant PEComa of the left acetabulum tried five cycles of temsirolimus with a favorable response of the suspected pulmonary metastases. However, the patient passed away eight months postoperatively despite several months of additional treatment [8].

## Conclusions

We have described the clinicopathological and immunohistochemical features of a malignant PEComa arising primarily in the femur of an adult male. Although very rare, PEComa can present as a primary bone lesion. Clinical and pathological correlation is mandatory in arriving at the correct diagnosis.

## Consent

Written informed consent was obtained from the patient for publication of this case report and any accompanying images. A copy of the written consent is available for review by the Editor of this journal.
